# Longitudinal assessment of coronary plaque regression related to sodium–glucose cotransporter-2 inhibitor using coronary computed tomography angiography

**DOI:** 10.1186/s12933-024-02368-y

**Published:** 2024-07-22

**Authors:** Tianhao Zhang, Xuelian Gao, Tianlong Chen, Hongkai Zhang, Xiaoming Zhang, Yu Xin, Dongmei Shi, Yu Du, Lei Xu, Yujie Zhou

**Affiliations:** 1 Department of Cardiology, Beijing Anzhen Hospital, Beijing Institute of Heart Lung and Blood Vessel Disease, Beijing Key Laboratory of Precision Medicine of Coronary Atherosclerotic Disease, Clinical center for coronary heart disease, Capital Medical University, Beijing, 100029 China; 2 Department of Radiology, Beijing Anzhen Hospital, Beijing Institute of Heart Lung and Blood Vessel Disease, Capital Medical University, Beijing, 100029 China; 3 Beijing Institute of Heart Lung and Blood Vessel Disease, Beijing, 100029 China

**Keywords:** Atherosclerotic plaque, Type 2 diabetes mellitus, Coronary computed tomography angiography, Sodium-Glucose Cotransporter-2 Inhibitor, Longitudinal study

## Abstract

**Background:**

Sodium–Glucose Cotransporter-2 Inhibitor (SGLT2i) is a novel oral drug for treating type 2 diabetes mellitus (T2DM) with demonstrated cardiovascular benefits. Previous studies in apolipoprotein E knockout mice have shown that SGLT2i is associated with attenuated progression of atherosclerosis. However, whether this effect extends to T2DM patients with coronary atherosclerosis in real-world settings remains unknown.

**Methods:**

In this longitudinal cohort study using coronary computed tomography angiography (CCTA), T2DM patients who underwent ≥ 2 CCTA examinations at our center between 2019 and 2022 were screened. Eligible patients had multiple study plaques, defined as non-obstructive stenosis at baseline and not intervened during serial CCTAs. Exclusion criteria included a CCTA time interval < 12 months, prior SGLT2i treatment, or initiation/discontinuation of SGLT2i during serial CCTAs. Plaque volume (PV) and percent atheroma volume (PAV) were measured for each study plaque using CCTA plaque analysis software. Patients and plaques were categorized based on SGLT2i therapy and compared using a 1:1 propensity score matching (PSM) analysis.

**Results:**

The study included 236 patients (mean age 60.5 ± 9.5 years; 69.1% male) with 435 study plaques (diameter stenosis ≥ 50%, 31.7%). Following SGLT2i treatment for a median duration of 14.6 (interquartile range: 13.0, 20.0) months, overall, non-calcified, and low-attenuation PV and PAV were significantly decreased, while calcified PV and PAV were increased (all *p* < 0.001). Meanwhile, reductions in overall PV, non-calcified PV, overall PAV, and non-calcified PAV were significantly greater in SGLT2i-treated compared to non-SGLT2i-treated plaques (all *p* < 0.001). PSM analysis showed that SGLT2i treatment was associated with higher reductions in overall PV (− 11.77 mm^3^ vs. 4.33 mm^3^, *p* = 0.005), non-calcified PV (− 16.96 mm^3^ vs. − 1.81 mm^3^, *p* = 0.017), overall PAV (− 2.83% vs. 3.36%, *p* < 0.001), and non-calcified PAV (− 4.60% vs. 0.70%, *p* = 0.003). These findings remained consistent when assessing annual changes in overall and compositional PV and PAV. Multivariate regression models demonstrated that SGLT2i therapy was associated with attenuated progression of overall or non-calcified PV or PAV, even after adjusting for cardiovascular risk factors, medications, and baseline overall or non-calcified PV or PAV, respectively (all *p* < 0.05). The effect of SGLT2i on attenuating non-calcified plaque progression was consistent across subgroups (all *p* for interaction > 0.05).

**Conclusions:**

In this longitudinal CCTA cohort of T2DM patients, SGLT2i therapy markedly regressed coronary overall PV and PAV, mainly result from a significant reduction in non-calcified plaque.

**Graphical abstract:**

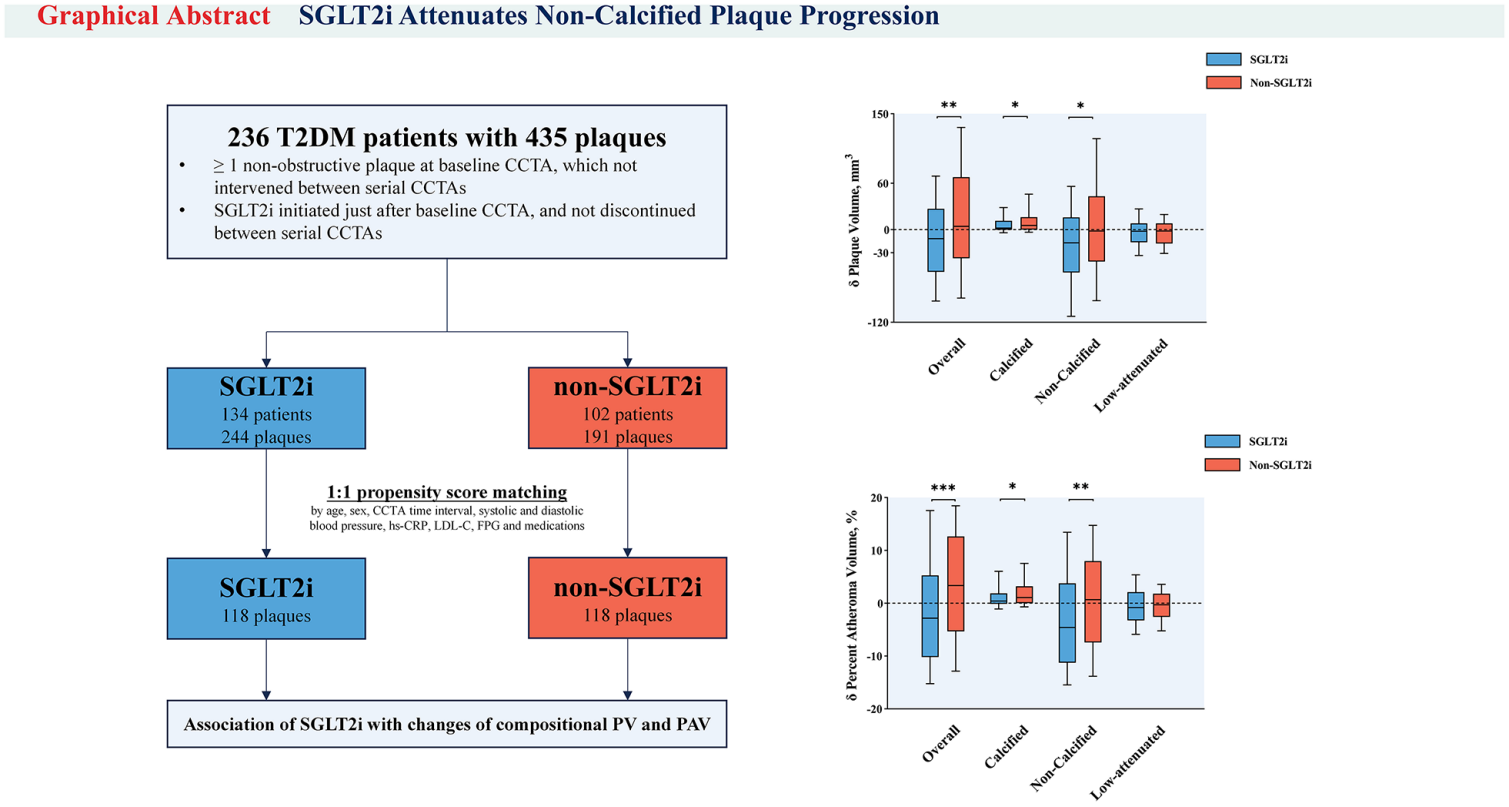

**Supplementary Information:**

The online version contains supplementary material available at 10.1186/s12933-024-02368-y.

## Introduction

According to the International Diabetes Federation, approximately 537 million individuals worldwide were affected by type 2 diabetes mellitus (T2DM) in 2021, with this number expected to rise to 783 million by 2045 [1]. Patients with T2DM develop atherosclerosis at a younger age and progress more rapidly compared to those without T2DM [2], thus increasing their risk of atherosclerotic cardiovascular disease (ASCVD) [3, 4]. While improved glycemic control with traditional glucose-lowering drugs has shown benefits in patients with newly diagnosed T2DM, these strategies are less effective in T2DM patients with established ASCVD, who face an elevated risk of premature cardiovascular events [5].

 Sodium-Glucose Cotransporter-2 Inhibitor (SGLT2i) is a novel oral hypoglycemic agent for the treatment of T2DM, which reduces glucose reabsorption by inhibiting SGLT2 in the renal proximal tubules [6]. The cardiovascular benefits of SGLT2i have been examined in six cardiovascular outcome trials (CVOTs) among T2DM patients [7–12]. A recent meta-analysis of these trials has demonstrated a significant reduction in the composite of cardiovascular death, myocardial infarction (MI), or stroke, particularly among patients with ASCVD [13]. Therefore, international guidelines recommend SGLT2i for patients with T2DM and ASCVD to reduce cardiovascular events, irrespective of glycosylated hemoglobin A1c levels and concomitant antidiabetic medications [14]. However, the ASCVD-based benefits of SGLT2i in T2DM patients remain unclear.

The pathological basis of ASCVD lies in the initiation and development of atherosclerotic plaque, which traditionally leads to MI or ischemic stroke. Preclinical studies have shown that SGLT2i can attenuate the progression of aortic plaque in apolipoprotein E knockout (ApoE^−/−^) mice [15, 16]. Additionally, SGLT2i therapy has been effective in stabilizing atherosclerotic plaque in a tandem stenosis ApoE^−/−^ mouse model [17]. Consistent with these findings, compared to non-SGLT2i treatment, SGLT2i was associated with a 9% reduction in MI in the aforementioned meta-analysis (95% confidence intervals [CI], 0.84–0.99) [13].

Therefore, the cardiovascular outcome benefits of SGLT2i may be attributed to the attenuation of plaque progression. However, this hypothesis has not been investigated among T2DM patients with coronary atherosclerosis in real-world settings. Importantly, the modification of coronary plaque response to medication can be accurately traced using high-resolution coronary computed tomography angiography (CCTA) and artificial intelligence-aided post-processing software [18]. Hence, we performed a longitudinal CCTA cohort study among T2DM patients with coronary atherosclerosis to evaluate the effects of SGLT2i on the progression of coronary atherosclerosis.

## Methods

### Study design and population

**Fig. 1:**
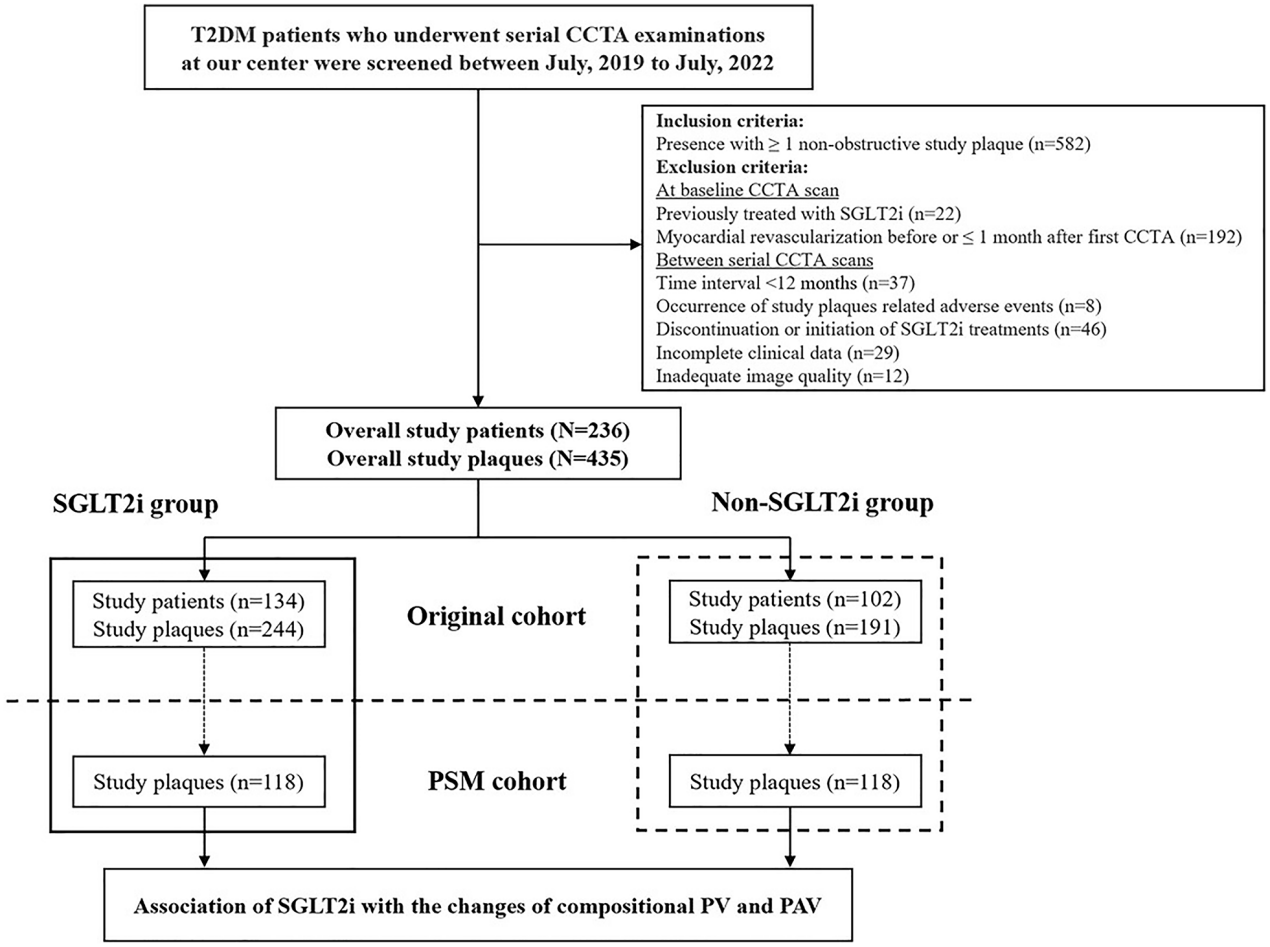
Flowchart of study process. *CCTA* coronary computed tomographic angiography, *PSM* propensity score matching, *PV* plaque volume, *PAV* percent atheroma volume, *SGLT2i* Sodium-Glucose Cotransporter-2 Inhibitor, *T2DM* type 2 diabetes mellitus

Patients diagnosed with T2DM who underwent at least two clinically indicated CCTA examinations at Beijing Anzhen Hospital, Capital Medical University, were screened between July 2019 to July 2022. Eligible T2DM patients had more than one study plaque, defined as non-obstructive plaque in one of the major coronary arteries. Exclusion criteria included: (1) Patients previously treated with SGLT2i before enrollment; (2) Patients who underwent myocardial revascularization before or within 1 month after the first CCTA; (3) Patients with a time interval between serial CCTA scans of less than 12 months(4) Patients with study plaque-related adverse events; (5) Patients who discontinued or initiated SGLT2i treatments during serial CCTA scans (6) Patients with incomplete clinical data and inadequate image quality. Study participants and plaques were categorized based on initiation of SGLT2i therapy within 1 month after the first CCTA scan. After propensity score matching (PSM), plaque progression was compared between SGLT2i and non-SGLT2i groups (Fig. 1). SGLT2i group received treatment of Dapagliflozin (5 mg daily), Empagliflozin (10 mg daily) or Canagliflozin (100 mg daily) at the first CCTA and follow-up. The study protocol was in accordance with the Declaration of Helsinki and was approved by the Medical Ethics Committee of Beijing Anzhen Hospital, with informed consent obtained from all participants.

### Data collection and follow-up

Demographic information, risk factors, medical histories, laboratory results, CCTA scan parameters, and medications were retrieved from electronic medical records. Adverse events and medications during longitudinal CCTA scans were monitored through outpatient or telephone interviews. Study plaque-related adverse events were defined as cardiac death, MI or revascularization during series CCTA follow-ups attributed to the study plaques.

### CCTA acquisition

All CCTA examinations were conducted using a 256-slice CT scanner (Revolution CT, GE Healthcare, USA) following the guidelines of the Society of Cardiovascular Computed Tomography [19]. A bolus of 40–60 mL of contrast media (Ultravist, Bayer) at a concentration of 370 mg iodine/mL was injected into the antecubital vein at a rate of 4–5 mL/s, followed by 30 mL of saline. CT scan parameters included a reconstructed layer thickness of 0.625 mm, a gantry rotation time of 0.28 s, as well as a tube voltage of 100 or 120 kV. The tube current was automatically adjusted using Smart-mA technology. Identical acquisition parameters were maintained for each patient at both baseline and follow-up CCTA scans.

### CCTA quantitative analysis

Anonymous CCTA datasets were transferred to an offline workstation for image quantitative analysis using semi-automated coronary plaque analysis software (Circle Cardiovascular Imaging, Canada, Version 5.13) with manual correction. Computed tomography-derived fractional flow reserve related to study plaque was calculated using an artificial intelligence-based automated analysis software (Shukun Technology, Beijing) [20]. CCTA images were analyzed by independent level-III experts who were blinded to the presence or absence of SGLT2i therapy and the order of serial CCTA scans.

**Fig. 2:**
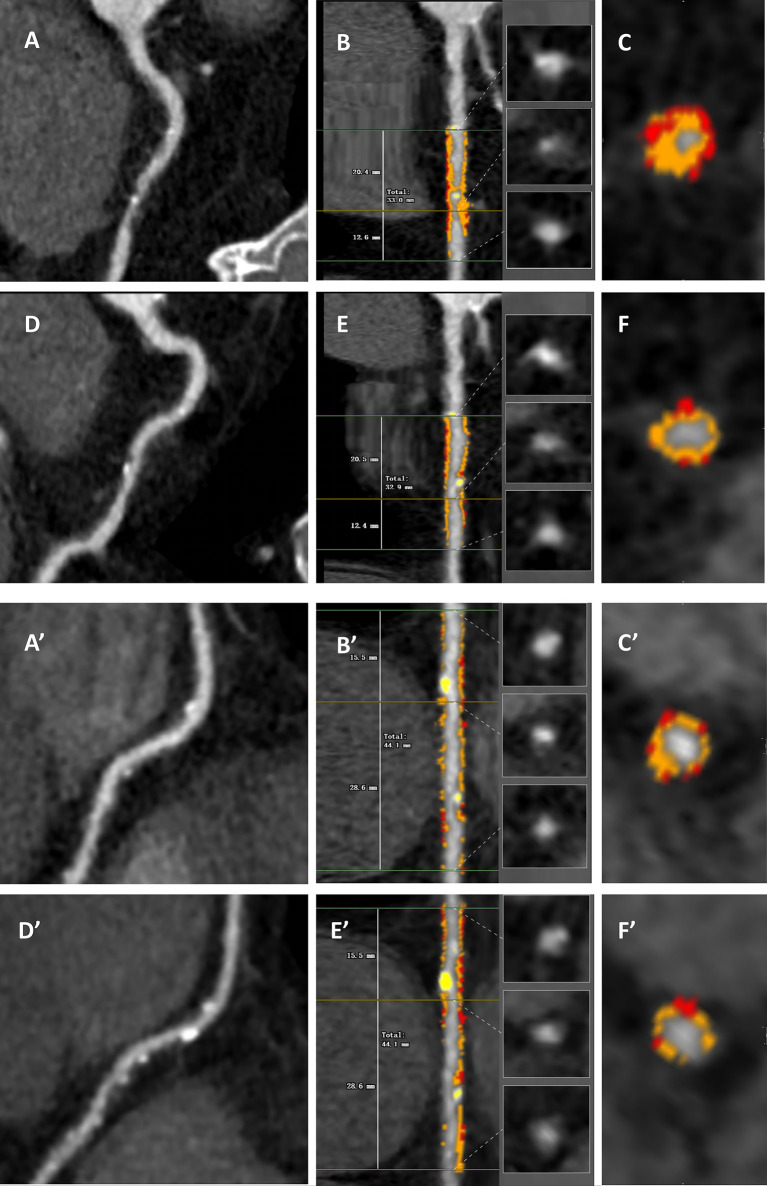
Representative patients treated with or without SGLT2i underwent longitudinal assessment of compositional plaque volume. **A**–**F** A 72 years old male with hypertension and hyperlipidemia (BMI 24.5 kg/m^2^, LDL-C 1.35 mmol/L, FPG 5.75 mmol/L) treated with SGLT2i, moderate intensity statin and ezetimibe. His baseline overall PV in the proximal right coronary artery (**A**) was 277.60 mm^3^ with non-calcified PV 276.06 mm^3^, low-attenuated PV 51.63 mm^3^ and calcified PV 1.54 mm^3^ (**B**, **C**). After 12.3 months, the overall PV in the proximal right coronary artery (**D**) decreased to 234.64 mm^3^ with non-calcified PV 232.86 mm^3^, low-attenuated PV 39.84 mm^3^ and calcified PV 1.78 mm^3^ (**E**, **F**). **A**’–**F**’ A 67 years old male with hypertension and hyperlipidemia (BMI 26.1 kg/m^2^, LDL-C 1.40 mmol/L, FPG 6.23 mmol/L) treated with moderate intensity statin and ezetimibe but without SGLT2i. His baseline overall PV in the middle right coronary artery (**A**’) was 185.27 mm^3^ with non-calcified PV 181.21 mm^3^, low-attenuated PV 34.78 mm^3^ and calcified PV 4.06 mm^3^ (**B**’, **C**’). After 15.7 months, the overall PV in the middle right coronary artery (**D**’) was 185.65 mm^3^ with non-calcified PV 178.21 mm^3^, low-attenuated PV 49.26 mm^3^ and calcified PV 7.44 mm^3^ (**E**’, **F**’). The orange, yellow and red overlays represent non-calcified, calcified and low-attenuated PV, espectively

Segments of major coronary arteries with a diameter ≥ 2 mm were evaluated based on a modified 17-segment model. The presence of atherosclerotic plaque was defined as any tissue exceeding 1  mm^3^ within or close to the lumen, which could be discriminated from surrounding structures and identified in more than 2 consecutive planes. Study plaques were confined to those with a diameter stenosis < 70% (i.e., non-obstructive) and not intervened. The 3D quantitative parameters included overall and compositional plaque volume (PV) and percent atheroma volume (PAV). Plaque composition was analyzed using the following Hounsfield unit (HU) thresholds: calcified (≥ 350 HU), non-calcified (< 350 HU), and low-attenuation (< 30 HU) [21–23]. For the longitudinal analysis of changes in PV and PAV between serial CCTA scans, coronary plaques were co-registered using consistent landmarks (e.g., distance from the ostium or the branch vessels). Representative serial cases of T2DM patients treated with or without SGLT2i were shown in Fig. 2.

To assess inter- and intra-observer variabilities, a second level-III reader re-analyzed 8 randomly selected plaques, and the original level-III reader re-analyzed 12 randomly selected plaques 3 months after the first analysis.

### Statistical analysis

Data were presented for the SGLT2i and non-SGLT2i groups on a per-patient level and a per-plaque level, respectively. Continuous variables were expressed as the mean ± standard deviation (SD) or medians with interquartile ranges (IQR), and compared between groups using the paired or unpaired Student’s t-test or Mann–Whitney U-test. Categorical variables were presented as frequencies (percentages) and compared between groups using the chi-square test or Fisher’s exact test.

To balance the differences in baseline characteristics between groups, a pre-specified 1:1 nearest-neighbor PSM analysis was performed on a per-plaque level using the following baseline variables: unbalanced variables between groups or variables might interfere with the clinical allocation of SGLT2i (blood pressure, fasting plasma glucose [FPG], stroke, CCTA time interval and medication), or well-known accelerators of atherosclerotic plaque (age, sex, blood pressure, FPG, high-sensitivity C-reactive protein [CRP], low-density lipoprotein cholesterol [LDL-C]). To account for variations in body habitus and CCTA time interval among study plaques, PAV (percentage of PV divided by vessel volume at each plaque) and annual changes of PV and PAV (changes of PV and PAV divided by CCTA interval years) were calculated, respectively. To further determine the association of SGLT2i with progression of compositional PV and PAV, multivariate regression analysis was performed. In the linear regression analysis, compositional PV and PAV were modeled as continual dependent variables, and results were shown as β and 95%CI. Meanwhile, in the logistic regression analysis, compositional PV and PAV were modeled as categorized dependent variables, and results were shown as odds ratios (ORs) and 95%CI. Independent variables with a *p*-value < 0.1 in the univariate analysis and known accelerators of atherosclerosis were included in the multivariate regression analysis.

Subgroup analysis was carried out to confirm whether the association of SGLT2i with PV or PAV progression was consistent across all pre-specified subgroups. Statistical analyses were conducted using SPSS 25.0 (IBM Corporation, IL, USA) as well as R Programming Language 4.2.2 (Vienna, Austria). Statistical significance was determined by *p* < 0.05 (two-tailed).

## Results

### Characteristics of study patients and plaques

In our initial analysis, 236 patients with T2DM were included, with a mean age of 60.5 ± 9.5 years, and 163 (69.1%) of them were male (Table 1). Among these patients, 68.2% had established ASCVD, while hypertension (73.7%), dyslipidemia (86.0%), and poor glucose control (median FPG of 7.5 mmol/L) were prevalent. Notably, patients treated with SGLT2i were more prone to have higher FPG levels and diastolic blood pressure (both *p* < 0.05) (Table 1). A total of 435 study plaques were included, with 31.7% exhibiting diameter stenosis ≥ 50%, and 25.1% having computed tomography-derived fractional flow reserve ≤ 0.80 (Table S1).

**Table 1:** Baseline characteristics of the overall study patients

	Total *n* = 236	SGLT2i *n* = 134	Non-SGLT2i *n* = 102	*p* value
Clinical characteristics				
Age, years	60.5 ± 9.5	60.1 ± 10.3	61.1 ± 8.4	0.402
Male, n (%)	163 (69.1)	95 (70.9)	68 (66.7)	0.486
BMI, kg/m^2^	26.0 (24.1, 28.7)	25.7 (24.0, 28.1)	26.2 (24.2, 28.8)	0.462
SBP, mmHg	131.5 (120.0, 140.0)	131.7 (118.0, 140.0)	131.5 (122.8, 141.0)	0.365
DBP, mmHg	76.0 (68.0, 84.0)	77.5 (69.0, 85.0)	74.0 (65.0, 81.3)	**0.033**
Risk factors, n (%)				
Hypertension	174 (73.7)	97 (72.4)	77 (75.5)	0.592
Dyslipidemia	203 (86.0)	116 (86.6)	87 (85.3)	0.780
Current smoking	73 (30.9)	45 (33.6)	28 (27.5)	0.313
Medical histories, n (%)				
Myocardial infarction	26 (11.0)	13 (9.7)	13 (12.7)	0.459
Myocardial revascularization	52 (22.0)	32 (23.9)	20 (19.6)	0.433
Stroke	33 (14.0)	16 (11.9)	17 (16.7)	0.300
Laboratory results				
FPG, mmol/L	7.5 (6.3, 9.2)	7.7 (6.6, 9.7)	7.1 (5.6, 8.6)	**0.009**
Creatinine, mmol/L	72.7 (63.5, 86.7)	72.9 (64.5, 87.0)	72.6 (62.6, 86.0)	0.771
eGFR, mL/min/1.73 m^2^	91.7 (78.9, 101.0)	91.4 (80.9, 101.5)	92.0 (76.8, 100.3)	0.799
TG, mmol/L	1.48 (1.09, 2.09)	1.48 (1.17, 2.10)	1.50 (1.06, 2.08)	0.421
TC, mmol/L	3.98 (3.41, 4.68)	3.93 (3.44, 4.68)	4.00 (3.38, 4.90)	0.646
HDL-C, mmol/L	1.01 (0.86, 1.19)	1.01 (0.86, 1.18)	1.02 (0.87, 1.24)	0.535
LDL-C, mmol/L	2.15 (1.67, 2.77)	2.14 (1.68, 2.76)	2.21 (1.66, 2.78)	0.758
hs-CRP, mg/L	1.15 (0.60, 2.51)	1.21 (0.59, 3.21)	1.15 (0.60, 2.32)	0.850
Serial CCTAs				
Time interval, month	14.6 (13.0, 20.0)	14.1 (12.9, 17.4)	15.9 (13.2, 23.0)	**0.030**
Baseline tube voltage, n (%)				0.840
100 kV	159 (67.4)	91 (67.9)	68 (66.7)	
120 kV	77 (32.6)	43 (32.1)	34 (33.3)	
Number of study plaque, per Patient	435 (1.84)	244 (1.82)	191 (1.87)	-
Medications, n (%)				
Metformin	95 (40.3)	47 (35.1)	48 (47.1)	0.063
Incretins	49 (20.8)	31 (23.1)	18 (17.6)	0.303
Insulin	48 (20.3)	26 (19.4)	22 (21.6)	0.682
Statins	236 (100)	134 (100)	102 (100)	1.000
Ezetimibe	61 (25.8)	37 (27.6)	24 (23.5)	0.478

*BMI* body mass index, *CCTA* coronary computed tomographic angiography, *DBP* diastolic blood pressure, *eGFR* estimated glomerular filtration rate, *FPG* fasting plasma glucose, *HDL-C* High density lipoprotein cholesterol, *hs-CRP* hypersensitive C-reactive protein, *LM* Left main artery, *LAD* left anterior descending artery, *LCX* left circumflex coronary artery, *LDL-C* low density lipoprotein cholesterol, *RCA* right coronary artery, *SGLT2i* Sodium-Glucose Cotransporter-2 Inhibitor, *SBP* systolic blood pressure, *TG* Triglyceride, *TC* Total cholesterol

**Table 2:** Baseline and follow-up CCTA findings on a per-plaque level

	Unmatched			Matched		
	SGLT2i	Non-SGLT2i	*p* value	SGLT2i	Non-SGLT2i	*p* value
	(*n* = 244)	(*n* = 191)		(*n* = 118)	(*n* = 118)	
Plaque volume, mm^3^						
Overall						
Baseline	221.69 (146.11, 328.02)	221.73 (154.99, 308.26)	0.958	220.50 (144.55, 292.13)	217.36 (161.95, 300.27)	0.894
Follow-up	215.06 (136.64, 301.24)	230.67(154.69, 335.58)	0.310	210.56 (138.72, 276.58)	218.49(156.28, 317.15)	0.129
Change from baseline	− 12.71 (− 62.16, 30.40)	2.92 (− 41.45, 65.18)	**< 0.001**	− 11.77 (− 54.58, 27.03)	4.33 (− 37.06, 68.07)	**0.005**
*p* value	**< 0.001**	0.073		**0.018**	**0.040**	
Calcified						
Baseline	5.94 (0.47, 28.16)	11.62 (1.94, 39.26)	**0.011**	6.02 (0.52, 21.71)	13.23 (2.26, 39.84)	**0.013**
Follow-up	9.98 (1.11, 34.36)	18.31 (3.30, 51.62)	**0.006**	11.75 (1.66, 24.85)	19.30 (3.72, 52.98)	**0.011**
Change from baseline	1.75 (− 0.45, 10.58)	3.45 (0.00, 15.65)	0.055	1.96 (− 0.11, 11.54)	5.38 (0.00, 16.15)	**0.033**
*p* value	**< 0.001**	**< 0.001**		**< 0.001**	**< 0.001**	
Non-calcified						
Baseline	202.92 (136.54, 280.27)	194.31 (128.35, 281.72)	0.533	198.17 (141.49, 263.09)	188.92 (127.93, 256.09)	0.447
Follow-up	177.26 (119.71, 264.13)	186.56 (127.68, 278.02)	0.082	175.84 (120.85, 242.35)	178.57 (126.01, 261.16)	0.610
Change from baseline	− 20.01 (− 68.31, 19.68)	− 2.78 (− 45.05, 44.60)	**< 0.001**	− 16.96 (− 55.51, 16.04)	− 1.81 (− 41.26, 43.27)	**0.017**
*p* value	**<0.001**	0.893		**0.001**	0.636	
Low-attenuated						
Baseline	29.86 (19.26, 51.73)	30.56 (19.32, 50.68)	0.832	27.51 (18.82, 48.47)	30.49 (19.43, 49.80)	0.739
Follow-up	26.76 (16.67, 42.79)	27.21 (17.27, 48.03)	0.602	27.04 (17.26, 38.89)	25.21 (17.77, 41.33)	0.757
Change from baseline	− 4.05 (− 16.66, 6.99)	− 2.14 (− 15.27, 8.92)	0.188	− 1.92 (− 16.33, 8.04)	− 1.82 (− 17.91, 8.18)	0.977
*p* value	**<0.001**	0.154		0.162	0.168	
Percent atheroma volume, %						
Overall						
Baseline	43.68 (35.71, 53.12)	40.20 (32.08, 50.25)	**0.030**	44.13 (37.14, 52.84)	40.01 (32.01, 52.57)	0.086
Follow-up	42.25 (34.64, 51.40)	42.98 (34.98, 52.04)	0.782	43.47 (35.25, 50.96)	44.21 (36.53, 54.27)	0.202
Change from baseline	− 2.25 (− 10.23, 6.77)	1.52 (− 6.31, 10.15)	**0.003**	− 2.83 (− 10.17, 5.27)	3.36 (− 5.31, 12.65)	**< 0.001**
*p* value	**0.047**	**0.028**		**0.043**	**0.003**	
Calcified						
Baseline	1.10 (0.09, 4.51)	2.15 (0.37, 6.15)	**0.010**	1.17 (0.09, 3.80)	2.15 (0.47, 6.58)	**0.020**
Follow-up	1.96 (0.19, 6.52)	3.57 (0.70, 8.09)	**0.008**	2.25 (0.36, 5.14)	3.62 (0.81, 9.46)	**0.018**
Change from baseline	0.21 (− 0.11, 1.96)	0.74 (0.00, 3.12)	**0.003**	0.41 (− 0.10, 1.88)	1.08 (0.00, 3.20)	**0.018**
*p* value	**< 0.001**	**< 0.001**		**< 0.001**	**< 0.001**	
Non-calcified						
Baseline	39.06 (30.55, 49.92)	36.18 (27.23, 46.87)	**0.025**	39.97 (33.02, 48.86)	35.81 (25.61, 47.63)	**0.024**
Follow-up	37.75 (27.58, 47.56)	36.09 (27.85, 45.41)	0.546	38.31 (27.65, 47.30)	37.16 (28.79, 47.04)	0.973
Change from baseline	− 3.88 (− 10.48, 4.38)	0.04 (− 7.73, 7.28)	**0.010**	− 4.60, (− 11.24, 3.78)	0.70 (− 7.42, 7.98)	**0.003**
*p* value	**< 0.001**	0.987		**0.002**	0.345	
Low-attenuated						
Baseline	5.93 (3.93, 9.17)	5.54 (3.56, 8.92)	0.355	5.89 (3.94, 9.51)	5.98 (3.60, 9.52)	0.613
Follow-up	5.36 (3.41, 8.80)	5.66 (3.22, 8.70)	0.937	5.40 (3.44, 9.30)	5.94 (3.29, 9.00)	0.829
Change from baseline	− 0.60 (− 2.77, 1.50)	− 0.33 (− 2.43, 1.86)	0.373	− 0.81 (− 3.24, 2.11)	− 0.30 (− 2.58, 1.82)	0.467
*p* value	**0.009**	0.252		**0.050**	0.271	
Diameter stenosis, %						
Baseline	41.8 (28.1, 55.3)	37.9 (23.1, 52.4)	**0.041**	41.6 (26.0, 54.6)	38.9 (25.4, 54.7)	0.716
Follow-up	39.2 (26.0, 51.6)	36.2 (25.5, 53.0)	0.501	40.0 (27.8, 53.6)	37.2 (27.1, 55.1)	0.526
Changes from baseline	− 1.5 (− 12.3, 9.0)	0.2 (− 11.6, 10.5)	0.330	0.8 (− 11.8, 9.1)	0.9 (− 10.6, 10.7)	0.870
*p* value	0.121	0.839		0.972	0.950	
FFR-CT						
Baseline	0.89 (0.81, 0.95)	0.88 (0.80, 0.93)	0.493	0.89 (0.82, 0.94)	0.87 (0.79, 0.93)	0.651
Follow-up	0.89 (0.81, 0.94)	0.89 (0.80, 0.94)	0.918	0.88 (0.81, 0.94)	0.87 (0.80, 0.93)	0.950
Changes from baseline	0.00 (− 0.04, 0.03)	0.00 (− 0.03, 0.03)	0.738	0.00 (− 0.04, 0.03)	0.00 (− 0.03, 0.03)	0.837
*p* value	0.419	0.779		0.459	0.590	

FFR-CT, computed tomography-derived fractional flow reserve, Other abbreviations shown in Table 1

### Temporal changes of overall and compositional PV and PAV

**Fig. 3:**
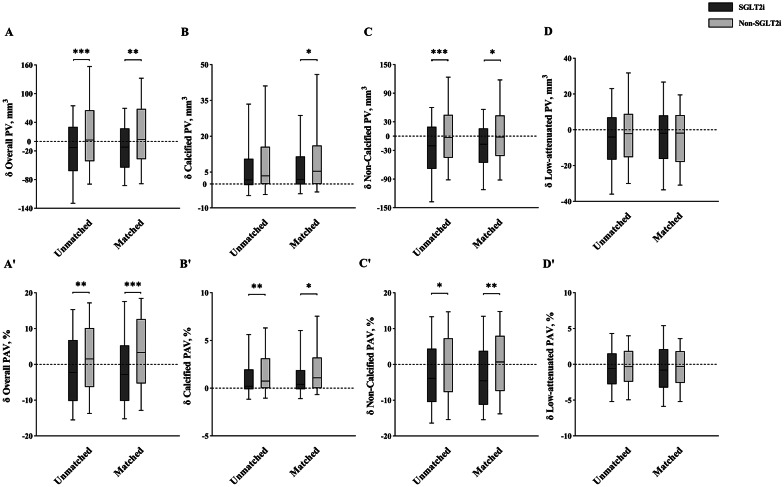
Temporal changes in compositional PV and PAV. *p < 0.05, **p < 0.01, ***p < 0.001. PV, plaque volume, PAV, percent atheroma volume

In our original analysis, after treatment with SGLT2i for a median duration of 14.6 (IQR: 13.0, 20.0) months, overall, non-calcified, and low-attenuation PV and PAV were significantly reduced, while calcified PV and PAV increased (all *p* < 0.001). However, these favorable plaque remodeling outcomes from SGLT2i did not translate into reductions in plaque-related anatomic and hemodynamic stenosis (all *p* > 0.05) (Table 2). Importantly, decreased overall PV (− 12.71 mm^3^ vs. 2.92 mm^3^, *p* < 0.001), non-calcified PV (− 20.01 mm^3^ vs. − 2.78 mm^3^, *p* < 0.001), overall PAV (− 2.25% vs. 1.52%, *p* = 0.003), and non-calcified PAV (− 3.88% vs. 0.04%, *p* = 0.010) were notably higher in SGLT2i-treated than non-SGLT2i-treated plaques (Table 2; Fig. 3).

The inter-observer variability of overall PV and PAV was 0.95 and 0.94, respectively, with corresponding intra-observer variability being 0.96 for both.

In the PSM analysis, baseline clinical and plaque characteristics were well-balanced between SGLT2i and non-SGLT2i groups (Table 2, Table S1). Similarly, compared to non-SGLT2i-treated plaques, those treated with SGLT2i showed a greater decrease in overall PV (− 11.77 mm^3^ vs. 4.33 mm^3^, *p* = 0.005), non-calcified PV (− 16.96 mm^3^ vs. − 1.81 mm^3^, *p* = 0.017), overall PAV (− 2.83% vs. 3.36%, *p* < 0.001), and non-calcified PAV (− 4.60% vs. 0.70%, *p* = 0.003), along with a lower increase in calcified PV (1.96 mm^3^ vs. 5.38 mm^3^, *p* = 0.033) and calcified PAV (0.41% vs. 1.08%, *p* = 0.018) (Table 2; Fig. 3).

### Annual changes of overall and compositional PV and PAV

When adjusting for differences in CCTA time interval, patients treated with SGLT2i consistently exhibited a higher decrease in overall PV (− 13.13 mm^3^/year vs. 2.28 mm^3^/year, *p* < 0.001), non-calcified PV (− 19.05 mm^3^/year vs. − 1.13 mm^3^/year, *p* < 0.001), overall PAV (− 2.38%/year vs. 1.26%/year, *p* = 0.002), and non-calcified PAV (− 3.47%/year vs. 0.02%/year, *p* = 0.002), while showing a lower increase in calcified PV (1.38 mm^3^/year vs. 2.47 mm^3^/year, *p* = 0.055) and calcified PAV (0.19%/year vs. 0.65%/year, *p* = 0.050). Importantly, these effects of SGLT2i on compositional PV and PAV remained unchanged in the PSM analysis (Table S2, Figure S1).

### Associations of SGLT2i with progression of overall and compositional PV and PAV

In a multivariate linear regression model, SGLT2i was negatively associated with overall (β = − 42.19, 95% CI − 63.13 to − 21.24; *p* < 0.001) and non-calcified (β = − 37.82, 95% CI − 57.85 to − 17.78; *p* < 0.001) PV progression, independent of age, sex, time interval, body mass index, diastolic blood pressure, current smoker status, LDL-C, high-sensitivity CRP, FPG, Metformin usage, Incretins, and baseline overall or non-calcified PV. When progression of overall or non-calcified PV was modeled as a binary variable, the negative effect of SGLT2i on the improvement of overall or non-calcified PV persisted (all *p* < 0.01). Additionally, SGLT2i improved the progression of overall and non-calcified PAV, whether PAV was modeled as a continuous or binary variable (all *p* < 0.05) (Table 3).

**Table 3:** Associations of SGLT2i with progression of compositional PV and PAV

	Adjusted variables*	Continuous plaque progression		Binary plaque progression	
		β (95%CI)	*p* value	OR (95%CI)	*p* value
Compositional PV					
Overall	+ Baseline overall PV	− 42.19 (− 63.13, − 21.24)	**< 0.001**	0.47 (0.31, 0.72)	**0.001**
Calcified	+ Baseline calcified PV	− 2.62 (− 8.28, 3.04)	0.364	0.66 (0.43, 1.03)	0.067
Non-calcified	+ Baseline non-calcified PV	− 37.82 (− 57.85, − 17.78)	**< 0.001**	0.51 (0.33, 0.78)	**0.002**
Low-attenuated	+ Baseline low-attenuated PV	− 5.39 (− 12.17, 1.40)	0.119	0.89 (0.58, 1.35)	0.572
Compositional PAV					
Overall	+ Baseline overall PAV	− 3.19 (− 5.37, − 1.01)	**0.004**	0.45 (0.28, 0.72)	**0.001**
Calcified	+ Baseline calcified PAV	− 0.60 (− 1.33, 0.13)	0.107	0.55 (0.36, 0.86)	**0.008**
Non-calcified	+ Baseline non-calcified PAV	− 2.57 (− 4.74, − 0.40)	**0.020**	0.51 (0.32, 0.80)	**0.004**
Low-attenuated	+ Baseline low-attenuated PAV	− 0.26 (− 1.01, 0.48)	0.488	0.82 (0.53, 1.26)	0.358

Variables in the basic model consisted of age, sex, time interval, BMI, DBP, current smoker, LDL-C, hs-CRP, FPG, Metformin, Incretins and SGLT2i

*Multivariate regression model adjusted for basic model + baseline compositional PV or PAV

### Subgroup analysis

**Fig. 4:**
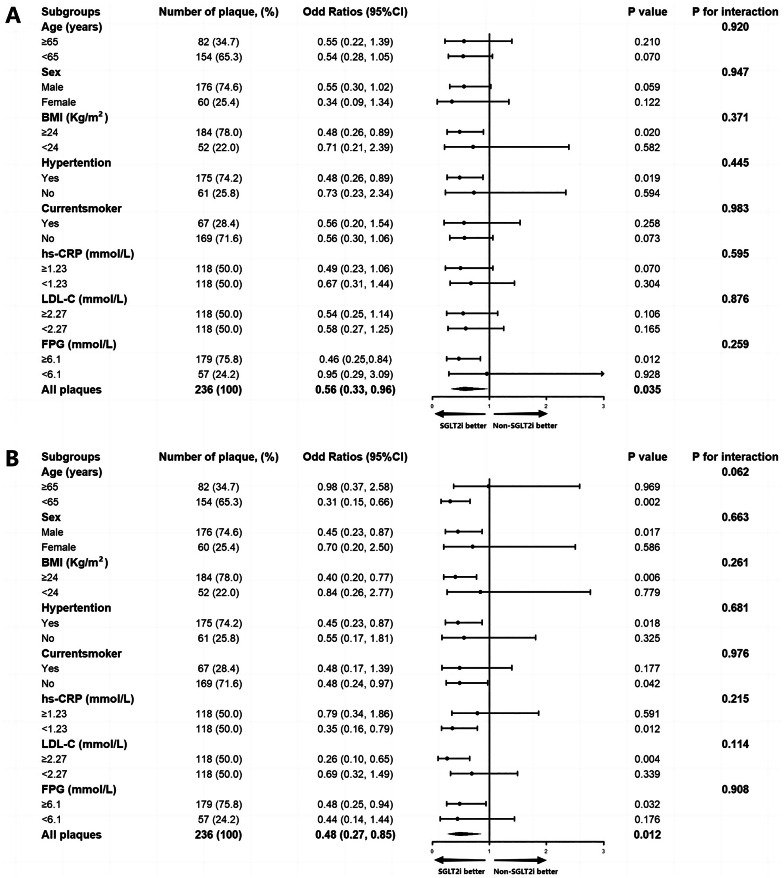
Subgroup analysis for the effect of SGLT2i on the progression of non-calcified PV and PAV. Subgroup analysis of the propensity score matching cohort for the effect of SGLT2i on the progression of non-calcified plaque volume (**A**) and percent atheroma volume (**B**). The black vertical solid line represents the OR value of 1. The subgroup analysis was adjusted for baseline non-calcified plaque volume or percent atheroma volume. *PV* plaque volume, *PAV* percent atheroma volume

To further elucidate the effect of SGLT2i on the progression of overall and non-calcified plaque, subgroup analysis of the PSM cohort was conducted based on age (< 65 or ≥ 65 years), sex (male or female), body mass index (< 24 or ≥ 24 kg/m^2^), hypertension (with or without), current smoker (yes or no), high-sensitivity CRP level (< 1.23 or ≥ 1.23 mmol/L), LDL-C (< 2.27 or ≥ 2.27 mmol/L), and FPG (< 6.1 or ≥ 6.1 mmol/L). After adjusting for baseline overall PV or PAV, no significant interactions between SGLT2i and overall PV or PAV were observed among subgroups, except for those with age < 65 favoring SGLT2i therapy (Figure S2). However, no significant interactions were detected among all subgroups between SGLT2i and non-calcified PV or PAV (all *p* for interaction > 0.05) (Fig. 4).

## Discussion

The main findings of this longitudinal CCTA cohort study were as follows: (1) SGLT2i therapy significantly reduced overall PV, primarily driven by a marked reduction of non-calcified PV, after following up for 14.6 months in median. (2) These findings were consistent in the PSM analysis between the SGLT2i and non-SGLT2i groups, in a sensitivity analysis with calculation of PAV and annualized change in PV, and in a multivariate model with adjustment of baseline PV or PAV. (3) Moreover, SGLT2i-treated plaques showed less increase in calcified PV and PAV compared to non-SGLT2i-treated plaques. (4) The effects of SGLT2i on optimal plaque remodeling were independent of age, sex, multiple cardiovascular risk factors, and co-existing medications. (5) However, the benefits of SGLT2i on plaque modification did not result in reductions of plaque-related anatomic and hemodynamic stenosis.

### Modification of atherosclerotic plaque

Growing evidence suggests that adverse cardiac events in patients with ASCVD are closely related to the atherosclerotic plaque itself rather than plaque-related anatomic and hemodynamic stenosis, known as *the plaque hypothesis* [24]. Therefore, stabilizing and regressing atherosclerotic plaque are primary objectives in ASCVD management. Previous trials have shown promising results in delaying plaque development using statins, evolocumab, low-dose colchicine, and icosapent ethyl [25–28]. However, the effect of SGLT2i on plaque progression among uncontrolled T2DM patients with ASCVD, where diabetes accelerates plaque progression, particularly in the non-calcified component, remains unclear [29].

### SGLT2i regresses atherosclerosis

A multicenter, randomized, open-label trial (Using Tofogliflozin for Possible better Intervention against Atherosclerosis for type 2 diabetes patients [UTOPIA]trial) evaluated the effect of tofogliflozin on preventing the development of carotid atherosclerosis among Japanese T2DM patients. Results indicated a significant reduction in carotid intima-media thickness (IMT) measured by ultrasonography at 2.2 and 4.3-year follow-ups, respectively. However, this reduction was not significantly different from conventional therapy [30, 31]. In contrast, our study comprehensively evaluated coronary plaque characteristics using high-resolution CCTA, which was more repeatable and less operator-dependent than ultrasonography. During a median follow-up of 14.6 months, we observed that traditional antidiabetic and antilipidemic therapies were not associated with decreased PV, whereas SGLT2i significantly reduced PV, particularly in SGLT2i-treated plaques, compared to non-SGLT2i-treated plaques. The differential effects of SGLT2i on plaque regression between our and UTOPIA trials may be attributed to the patient population, with our study comprising two-thirds of patients with established ASCVD. Additionally, CCTA provides more accurate and repeatable measurements of plaque features compared to ultrasonography. Importantly, we have systematically controlled the process of serial CCTA acquisition, and inter- and intra-observer variabilities of CCTA quantitative analysis were optimal.

### SGLT2i modifies plaque composition

In a sub-analysis of the UTOPIA trial, neither tofogliflozin nor conventional medications reduced the ultrasonic gray-scale median of carotid atherosclerosis, which comprises lipids, inflammatory infiltrations, and/or hemorrhages [32]. However, our study demonstrated a marked decrease in overall PV, predominantly driven by non-calcified PV, a component associated with adverse cardiovascular events [33]. Recent data from the Scottish Computed Tomography of the Heart (SCOT-HEART) trial highlighted the prognostic significance of low-attenuation non-calcified PV [34]. Although the reduction of low-attenuation PV by SGLT2i was not statistically significant, it might be masked by moderate-intensity statin therapy, which was prescribed to each patient of the current study. In line with this hypothesis, calcified PV consistently increased independently of SGLT2i treatment [25].

To further investigate the effect of SGLT2i on plaque composition and vulnerability, intracoronary optical coherence tomography, with image resolution of ~ 10 μm, was used. Sardu et al. investigated 369 T2DM patients with multivessel non-obstructive coronary stenosis and found that SGLT2i treatment was associated with a lower burden of lipid and macrophage-rich plaque and a thicker minimal fibrous cap at a 1-year follow-up [35]. Similarly, among 109 T2DM patients with co-existing acute coronary syndrome, Kurozumi et al. demonstrated that 6-month treatment with SGLT2i significantly improved the thickness of fibrous cap and reduced the total lipid arc [36]. These findings suggest that SGLT2i may decrease the vulnerable components of plaque and enhance plaque stabilization.

### Insights from pre-clinical studies

In the nature history of atherosclerotic plaque progression, monocyte-macrophage axis and relevant inflammatory pathways play a vital role. Dapagliflozin and Ipragliflozin were found to suppress macrophage polarization and macrophage foam cell formation in streptozotocin-induced diabetic ApoE^−/−^ mice [37]. In ApoE^−/−^ mice fed a western diet, Empagliflozin or Canagliflozin reduced aortic arch plaque and levels of pro-inflammatory cytokines, such as tumor necrosis factor-α, interleukin-6, monocyte chemoattractant protein-1, and vascular cell adhesion molecule-1 in the circulation or plaque [15, 16]. Empagliflozin reduced CD68^+^ macrophages and lipid content, while increased collagen content in the atherosclerotic plaque of diabetic mice [38]. Similarly, Dapagliflozin treatment in a dedicated vulnerable plaque model of ApoE^−/−^ mice induced collagen accumulation and fibrosis, increased cap-to-plaque height ratio, and elevated nicotinamide adenine dinucleotide oxidase 4 expression, indicating improved plaque stability [17].

Meanwhile, SGLT2i also reduced atheroma burden and lipid accumulation accompanied by suppression of Toll-like receptor 4/nuclear factor-kappa B signaling pathway, and their downstream inflammatory effectors in normoglycemic rabbit model [39]. Moreover, SGLT2i was also proved to reduce leukocyte adhesion and endothelial dysfunction that may contribute to plaque regression [38, 40].

Besides the benefits of SGLT2i on non-calcified plaque composition, it might reduce vascular calcification. Canagliflozin or Empagliflozin treatment prevented aortic calcification in mice through downregulating the expression of nucleotide-binding domain, leucine-rich-containing family, pyrin domain-containing-3 signaling pathway [41, 42]. In addition, Dapagliflozin reduced vascular calcification through blocking endoplasmic reticulum stress-dependent thioredoxin domain containing 5 upregulation and promoting subsequent runt-related transcription factor-2 proteasomal degradation [43].

### Study limitations

There were certain limitations in this study. Firstly, as a single-center study with a relatively small sample size, its generalizability may be limited. However, the study might provide valuable insights into the real-world use of SGLT2i among high-risk patients with T2DM in a tertiary hospital setting in China. Secondly, although we have performed thoughtful analysis to robustly ascertain the main findings, unadjusted variables may still exist, potentially affecting the link between SGLT2i and plaque regression (e.g., blood glucose control, other diabetes medications). Meanwhile, the retrospective nature of the study precludes establishing causality between SGLT2i and plaque regression, necessitating confirmation through dedicated prospective studies. Therefore, while the study generates hypotheses, caution should be exercised in generalizing the findings to other racial or ethnic populations. Thirdly, serial follow-up lab indices (e.g., hemoglobin A1c) or blood samples were lacking, precluding us to further robust and investigate mechanism of SGLT2i treatment effects. Lastly, a more comprehensive CCTA assessment of coronary plaque using advanced post-processing techniques is warranted, encompassing high-risk features, pericoronary inflammation, and biomechanical characteristics.

## Conclusions

In this longitudinal CCTA cohort study involving T2DM patients, SGLT2i therapy significantly regressed coronary overall PV and PAV, primarily driven by a marked reduction in the non-calcified plaque component. These findings offer insights into the potential mechanisms underlying the observed cardioprotective effects of SGLT2i in previous CVOTs.

### Electronic supplementary material

Below is the link to the electronic supplementary material.

Supplementary Material 1

## Data Availability

No datasets were generated or analysed during the current study.

## Abbreviations

ASCVD: Atherosclerotic cardiovascular disease
ApoE^−/−^: Apolipoprotein E knockout
CVOT: Cardiovascular outcome trials
CI: Confidence intervals
CCTA: Coronary computed tomography angiography
HU: Hounsfield unit
IQR: Interquartile ranges
IMT: Intima-media thickness
ORs: Odd ratios
MI: Myocardial infarction
PV: Plaque volume
PAV: Percent atheroma volume
PSM: Propensity score matching
SGLT2i: Sodium–Glucose Cotransporter-2 Inhibitor
T2DM: Type 2 diabetes mellitus
UTOPIA trial: Using Tofogliflozin for Possible better Intervention against Atherosclerosis for type 2 diabetes patients trial
