# Supplementary material for: Longitudinal assessment of coronary plaque regression related to sodium–glucose cotransporter-2 inhibitor using coronary computed tomography angiography
Source: Cardiovasc Diabetol. 2024 Jul 22;23:267. doi: 10.1186/s12933-024-02368-y (PMC11264370; doi:10.1186/s12933-024-02368-y)
Supplement: Supplementary file 1 — Supplementary Material 1 [file 12933_2024_2368_MOESM1_ESM.docx]

**SUPPLEMENTS**

**Longitudinal Assessment of** **Coronary Plaque Regression Related to Sodium-Glucose Cotransporter-2 Inhibitor Using Coronary Computed Tomography Angiography**

*Running title: SGLT2i and coronary plaque regression*

Tianhao Zhang^1^, Xuelian Gao^2^, Tianlong Chen^3^, Hongkai Zhang^2^, Xiaoming Zhang^1^, Yu Xin^1^, Dongmei Shi^1^, Yu Du^1*^, Lei Xu^2*^, Yujie Zhou^1*^

**Supplemental Table S1. Baseline characteristics of the overall study plaques**

|  |  | | | **Unmatched** | | | |  |  |  | **Matched** |  |
| --- | --- | --- | --- | --- | --- | --- | --- | --- | --- | --- | --- | --- |
|  | SGLT2i | | | Non-SGLT2i | | | | *p* value |  | SGLT2i | Non-SGLT2i | *p* value |
|  | (n=244) | | | (n=191) | | | |  |  | (n=118) | (n=118) |  |
| **Clinical characteristics** |  |  |  | |  |  |  |  |  |  |  |  |
| Age, years | 60.2±10.1 | | | 61.3±8.4 | | | | 0.225 |  | 60.4±10.1 | 60.1±8.4 | 0.839 |
| Male, n (%) | 173 (70.9) | | | 124 (64.9) | | | | 0.184 |  | 86 (72.9) | 90 (76.3) | 0.550 |
| BMI, kg/m^2^ | 25.9 (24.0, 28.2) | | | 26.3 (24.2, 28.7) | | | | 0.373 |  | 26.0 (24.0, 28.1) | 26.4 (24.3, 29.1) | 0.137 |
| SBP, mmHg | 131.2 (118.0, 139.8) | | | 132.0(124.0, 141.0) | | | | **0.031** |  | 132.0 (120.0, 140.0) | 130.0(120.0, 138.5) | 0.476 |
| DBP, mmHg | 78.0 (69.0, 85.0) | | | 76.0 (66.0, 83.0) | | | | **0.014** |  | 77.0 (69.0, 81.3) | 77.0 (67.0, 85.0) | 0.919 |
| **Risk factors, n (%)** |  | | |  | | | |  |  |  |  |  |
| Hypertension | 178 (73.0) | | | 147 (77.0) | | | | 0.339 |  | 91 (77.1) | 84 (71.2) | 0.298 |
| Dyslipidemia | 218 (89.3) | | | 163 (85.3) | | | | 0.209 |  | 103 (87.3) | 102 (86.4) | 0.847 |
| Current Smoking | 85 (34.8) | | | 51 (26.7) | | | | 0.069 |  | 32 (27.1) | 35 (29.7) | 0.665 |
| **Medical histories, n (%)** |  | | |  | | | |  |  |  |  |  |
| Myocardial infarction | 20 (8.2) | | | 19 (9.9) | | | | 0.526 |  | 13 (11.0) | 16 (13.6) | 0.552 |
| Myocardial revascularization | 50 (20.5) | | | 31 (16.2) | | | | 0.257 |  | 27 (22.9) | 26 (22.0) | 0.876 |
| Stroke | 32 (13.1) | | | 39 (20.4) | | | | **0.041** |  | 11 (9.3) | 17 (14.4) | 0.227 |
| **Lab tests** |  | | |  | | | |  |  |  |  |  |
| FPG, mmol/L | 7.6 (6.4, 9.6) | | | 6.9 (5.6, 8.6) | | | | **<0.001** |  | 7.5 (6.3, 8.7) | 7.6 (5.7, 9.2) | 0.831 |
| Creatinine, mmol/L | 72.5 (63.9, 87.0) | | | 72.7 (63.5, 86.4) | | | | 0.759 |  | 75.5 (64.7, 87.0) | 72.9 (63.5, 84.5) | 0.528 |
| eGFR, mL/min/1.73 m^2^ | 91.6 (81.5, 101.5) | | | 90.5 (76.2, 100.1) | | | | 0.182 |  | 91.8 (78.5, 99.9) | 94.8 (79.0, 101.1) | 0.376 |
| TG, mmol/L | 1.51 (1.17, 2.09) | | | 1.55 (1.07, 2.10) | | | | 0.436 |  | 1.45 (1.17, 1.96) | 1.40 (1.06, 1.98) | 0.408 |
| TC, mmol/L | 3.95 (3.43, 4.65) | | | 4.11 (3.39, 4.93) | | | | 0.319 |  | 4.18 (3.44, 4.81) | 4.11 (3.38, 5.01) | 0.868 |
| HDL-C, mmol/L | 1.01 (0.87, 1.19) | | | 1.01 (0.87, 1.25) | | | | 0.418 |  | 1.06 (0.89, 1.19) | 1.02 (0.87, 1.26) | 0.856 |
| LDL-C, mmol/L | 2.10 (1.65, 2.75) | | | 2.27 (1.72, 2.81) | | | | 0.235 |  | 2.27 (1.90, 2.84) | 2.24 (1.73, 2.90) | 0.889 |
| hs-CRP, mg/L | 1.09 (0.58, 3.07) | | | 1.23 (0.68, 2.34) | | | | 0.349 |  | 1.02 (0.49, 3.10) | 1.42 (0.68, 2.40) | 0.122 |
| **Time interval between CCTAs, month** | 14.2 (12.9, 17.8) | | | 17.6 (13.3, 23.7) | | | | **<0.001** |  | 14.2 (12.7, 21.5) | 15.0 (12.7, 23.2) | 0.605 |
| **Tube voltage of CT acquisition, n**  **(%)** |  | | |  | | | | 0.993 |  |  |  | 0.575 |
| 100 kV | 170 (69.7) | | | 133 (69.6) | | | |  |  | 83 (70.3) | 79 (66.9) |  |
| 120 kV | 74 (30.3) | | | 58 (30.4) | | | |  |  | 35 (29.7) | 39 (33.1) |  |
| **Study plaque*** |  | | |  | | | |  |  |  |  |  |
| Number, per patient | 1.82 (244/134) | | | 1.87 (191/102) | | | | - |  | 1.64(118/72) | 1.69(118/70) | - |
| Location, n (%) |  | | |  | | | | 0.533 |  |  |  | 0.988 |
| LAD | 76 (31.1) | | | 56 (29.3) | | | | - |  | 37 (31.4) | 36 (30.5) | - |
| LCX | 91 (37.3) | | | 65 (34.0) | | | | - |  | 44 (37.3) | 45 (38.1) | - |
| RCA | 77 (31.6) | | | 70 (36.6) | | | | - |  | 37 (31.4) | 37 (31.4) | - |
| Diameter stenosis ≥ 50%, n (%) | 85 (34.8) | | | 53 (27.7) | | | | 0.115 |  | 43 (36.4) | 39 (33.1) | 0.584 |
| FFR-CT ≤ 0.8, n (%) | 60(24.6) | | | 49(25.7) | | | | 0.799 |  | 28(23.7) | 35(29.7) | 0.303 |
| **Medications, n (%)** |  | | |  | | | |  |  |  |  |  |
| Metformin | 93 (38.1) | | | 90 (47.1) | | | | 0.059 |  | 47 (39.8) | 53 (44.9) | 0.429 |
| Incretins | 63 (25.8) | | | 31 (16.2) | | | | **0.016** |  | 29 (24.6) | 25 (21.2) | 0.535 |
| Insulin | 49 (20.1) | | | 41 (21.5) | | | | 0.724 |  | 27 (22.9) | 28 (23.7) | 0.878 |
| Statins | 244 (100) | | | 191 (100) | | | | 1.000 |  | 118 (100) | 118 (100) | 1.000 |
| Ezetimibe | 69 (28.3) | | | 43 (22.5) | | | | 0.172 |  | 34 (28.8) | 36 (30.5) | 0.776 |

BMI, body mass index; CCTA, coronary computed tomographic angiography; DBP, diastolic blood pressure; eGFR, estimated glomerular filtration rate; FPG, fasting plasma glucose; HDL-C, High density lipoprotein cholesterol; hs-CRP, hypersensitive C-reactive protein; LM, Left main artery: LAD, left anterior descending artery; LCX, left circumflex coronary artery; LDL-C, Low density lipoprotein cholesterol; RCA, right coronary artery; SGLT2i, Sodium-dependent glucose transporters 2 inhibitor; SBP, systolic blood pressure; TG, Triglyceride; TC, Total cholesterol

**Supplemental Table S2. Annual changes of compositional PV and PAV on a per-plaque level**

|  | **Unmatched** | | |  | **Matched** | | |
| --- | --- | --- | --- | --- | --- | --- | --- |
|  | SGLT2i | Non-SGLT2i | *p* value |  | SGLT2i | Non-SGLT2i | *p* value |
|  | (n=244) | (n=191) |  |  | (n=118) | (n=118) |  |
| **Annual PV change, mm^3^/year** |  |  |  |  |  |  |  |
| Overall | -13.13 (-61.35, 27.42) | 2.28 (-25.98, 51.75) | **<0.001** |  | -9.80 (-45.01, 20.62) | 3.28 (-28.45, 60.17) | **0.007** |
| Calcified | 1.38 (-0.43, 9.42) | 2.47 (0.00, 13.75) | 0.055 |  | 1.68 (-0.08, 10.01) | 4.83 (0.00, 16.26) | **0.024** |
| Non-Calcified | -19.05 (-66.39, 21.76) | -1.13 (-31.01, 40.29) | **<0.001** |  | -14.79 (-50.20, 18.28) | -0.94 (-32.27, 42.52) | **0.021** |
| Low-attenuated | -3.62 (-16.62, 6.40) | -1.77 (-11.41, 7.83) | 0.188 |  | -1.57 (-13.41, 7.98) | -1.98 (-13.25, 7.53) | 0.994 |
| **Annual PAV change, %/year** |  |  |  |  |  |  |  |
| Overall | -2.38 (-9.18, 6.26) | 1.26 (-4.77, 7.49) | **0.002** |  | -2.71 (-9.65, 3.76) | 2.56 (-4.34, 11.32) | **0.001** |
| Calcified | 0.19 (-0.11, 1.81) | 0.65 (0.00, 2.45) | **0.050** |  | 0.32 (-0.12, 1.63) | 0.89 (0.00, 3.33) | **0.018** |
| Non-Calcified | -3.47 (-9.90, 4.49) | 0.02 (-5.59, 6.10) | **0.002** |  | -4.13 (-10.59, 2.96) | 0.59 (-5.69, 6.99) | **0.002** |
| Low-attenuated | -0.48 (-2.70, 1.54) | -0.26 (-1.87, 1.36) | 0.204 |  | -0.76 (-2.71, 1.96) | -0.13 (-2.59, 1.42) | 0.476 |

Annualized change of PV or PAV indicated change of PV or PAV from baseline divided by the CCTA time intervals (years)

Abbreviations: PV, plaque volume; PAV, percent atheroma volume; Other abbreviations shown in Table S1.

**Supplemental Figure S1. Annual changes of compositional PV and PAV**


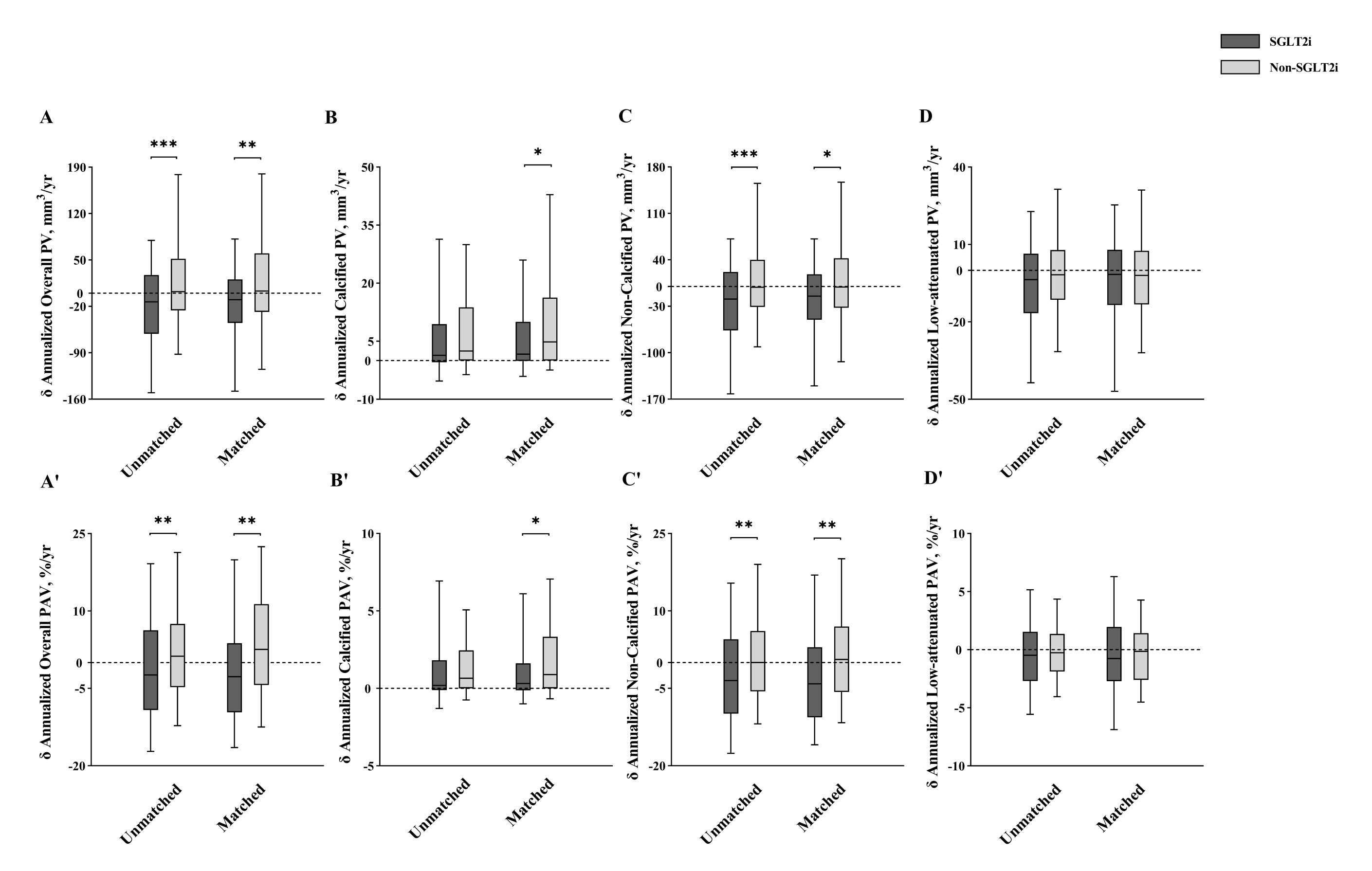


*p < 0.05, **p < 0.01, ***p < 0.001. Abbreviations: PV, plaque volume; PAV, percent atheroma volume; SGLT2i, Sodium-dependent glucose transporters 2 inhibitor

**Supplemental Figure S2. Subgroup analysis for the effect of SGLT2i on the progression of overall PV and PAV**


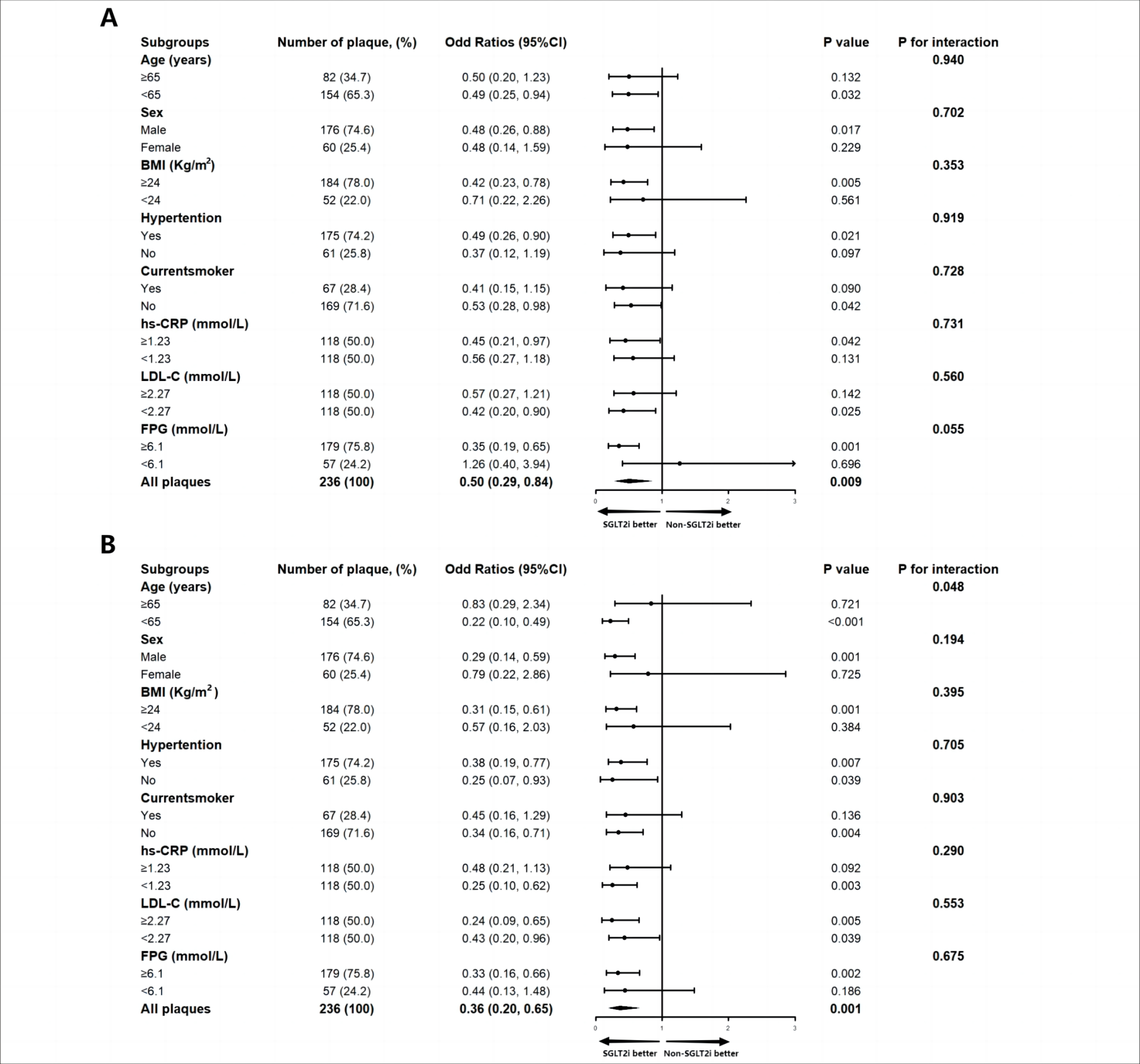


Subgroup analysis of the propensity score matching cohort for the effect of SGLT2i on the progression of overall plaque volume (A) and percent atheroma volume (B). The black vertical solid line represents the OR value of 1. The subgroup analysis was adjusted for baseline overall plaque volume or percent atheroma volume.

Abbreviations shown in Figure 1.
